# Maternal Adverse Childhood Experiences and Delayed Initiation of Complementary Foods: A Nationwide Online Cohort Study

**DOI:** 10.3390/nu17172879

**Published:** 2025-09-05

**Authors:** Yousuke Imanishi, Ichiro Wada, Sinchul Jwa, Mai Uchida, Takahiro Tabuchi

**Affiliations:** 1 Division of General Internal Medicine and Health Services Research, David Geffen School of Medicine at UCLA, Los Angeles, CA 90024, USA; 2 Department of Interdisciplinary Studies, Faculty of International Liberal Arts, Dokkyo University, Saitama 340-0042, Japan; i-wada@dokkyo.ac.jp; 3 Department of Pediatrics, Osaka Metropolitan University Graduate School of Medicine, Osaka 545-8585, Japan; sinchul.jwa@gmail.com; 4 Department of Psychiatry, Harvard Medical School, Boston, MA 02115, USA; muchida@mgb.org; 5 Department of Psychiatry, Massachusetts General Hospital, Boston, MA 02114, USA; 6 Division of Epidemiology, School of Public Health, Tohoku University Graduate School of Medicine, Sendai 980-8578, Japan; tabuchitak@gmail.com

**Keywords:** adverse childhood experiences, infant feeding, complementary feeds, formula feeding, postpartum depression, mediation, effect modification, inverse probability of treatment weighting, Japan

## Abstract

**Background/Objectives:** Infant feeding is critical for health and development, yet the influence of maternal psychosocial factors on its timing is not fully understood. While maternal adverse childhood experiences (ACEs) are known to affect perinatal outcomes, their impact on the specific timing of initiating formula and complementary foods remains under-investigated. We hypothesized that maternal ACEs are associated with delayed initiation of infant formula and complementary foods and that this association is mediated by postpartum depression (PPD). This study aimed to examine the link between maternal ACEs and delayed infant feeding, and to assess the mediating role of PPD using data from a large nationwide Japanese database. **Methods:** This cross-sectional study utilized data from the Japan COVID-19 and Society Internet Survey (JACSIS), conducted between July and August 2021. The analysis included 3446 postpartum mothers. Maternal ACEs were assessed using a 9-item questionnaire, and a cumulative score was categorized as high (≥4 ACEs) versus low (0–3 ACEs). The primary outcomes were infant feeding behavior including breastfeeding, formula feeding and complementary foods. We used logistic regression analysis with inverse probability of treatment weighting (IPTW) to calculate adjusted odds ratios (ORs) and 95% confidence intervals (CIs). A mediation analysis was conducted to evaluate the role of smoking, alcohol and PPD. **Results:** High ACE exposure (≥4) was present in 221 mothers (6.4%). A high maternal ACE score was significantly associated with delayed initiation of formula feeding (≥7 days) (Adjusted OR: 2.12, 95% CI: 1.12–4.01, *p* = 0.02) and late initiation of complementary foods (≥7 months) (Adjusted OR: 2.27, 95% CI: 1.38–5.01, *p* = 0.03); no significant associations were observed for ever/late/continued breastfeeding or ever/continued formula feeding. These associations attenuated to non-significance after adjusting for PPD. **Conclusions:** Maternal ACEs are associated with delayed initiation of complementary foods and formula, largely through PPD. Perinatal services should combine ACE/PPD screening with trauma-informed mental health and nutrition support to promote timely infant feeding.

## 1. Introduction

Infant feeding, which includes breastfeeding, formula feeding, and the introduction of complementary foods, is foundational to an infant’s short- and long-term health, growth, and development, making the timing of its initiation critically important [1,2]. However, individual differences exist in the timing of feeding initiation, and it is suggested that maternal psychosocial factors may play a significant role. Among these factors, maternal adverse childhood experiences (ACEs) are widely recognized to have a lifelong impact on adult mental and physical health, as well as on health-related behaviors [3,4]. Clarifying how maternal ACEs affect not only perinatal complications but also postpartum parenting behaviors—particularly the specific act of infant feeding—is essential for the early identification of at-risk postpartum mothers and the provision of targeted support [4].

Previous research has consistently reported an association between maternal ACEs and adverse perinatal outcomes, including preterm birth, low birth weight, and neonatal intensive care unit (NICU) admission [5,6]. It has also been shown that mothers who have experienced a high number of ACEs are at an increased risk of developing postpartum depression (PPD) [7] and are more likely to engage in health-risk behaviors such as smoking [4]. Postpartum depression, in turn, has been suggested to potentially reduce a mother’s engagement and confidence in parenting, thereby affecting mother-infant bonding and subsequent parenting behaviors [8].

Although research on the impact of ACEs on biological perinatal outcomes has advanced, how they affect the maternal “behavior” of infant feeding—specifically the initiation timing of formula and complementary foods—remains insufficiently understood. Furthermore, if ACEs do influence infant feeding practices, the underlying mechanisms, particularly the extent to which mental health challenges like PPD explain or mediate this relationship, require further investigation [7]. The reporting rates of ACEs and their underlying sociocultural contexts have been noted to vary by country and region. In fact, ACE reporting rates in the present study population differ from those in U.S.-based surveys [9]. Therefore, examining the association between maternal ACEs and infant feeding behaviors in a population with a different cultural background is crucial for assessing the generalizability of findings and deepening the universal understanding of this issue. While prior studies mainly address breastfeeding initiation/exclusivity in relation to maternal ACEs, evidence on the timing of formula or complementary foods is sparse, particularly in Asian settings. Our study extends this literature by focusing on delayed initiation thresholds (≥7 days for formula; ≥7 months for complementary foods) in a nationwide Japanese sample, thereby testing generalizability beyond Western countries [10,11,12].

In this study, we hypothesized that maternal ACEs are associated with delayed initiation of infant formula and complementary foods and that this association is mediated by postpartum mental health challenges. Accordingly, we aimed to examine whether maternal ACEs and delayed initiation of infant formula and complementary foods and whether postpartum depression mediates this association. We addressed this aim in a cross-sectional analysis of a large nationwide online survey in Japan.

## 2. Materials and Methods

### 2.1. Study Sample and Recruitment

This cross-sectional study was based on data from the Japan COVID-19 and Society Internet Survey (JACSIS), a comprehensive, nationwide research initiative established in Japan to assess the complex health and social consequences of the COVID-19 pandemic. The project operates through several online survey panels that target various demographics, including youth and adults, pregnant and postpartum mothers with their children, and adults in single-parent households. A more detailed account of the JACSIS methodology, quality assurance protocols, and panelist policies has been provided in previous publications [13].

For this present study on maternal and child health, the sample was derived from the JACSIS panel dedicated to pregnant and postpartum mothers. This panel recruits individuals from the large subscriber database of Rakuten Insight, Inc., a leading Japanese internet research firm that managed a panel of approximately 2.3 million people at the time of the survey. The panel was constructed to be nationally representative in its age and geographic distributions. Data were gathered from all 47 prefectures in Japan between July and August 2021.

**Figure 1:**
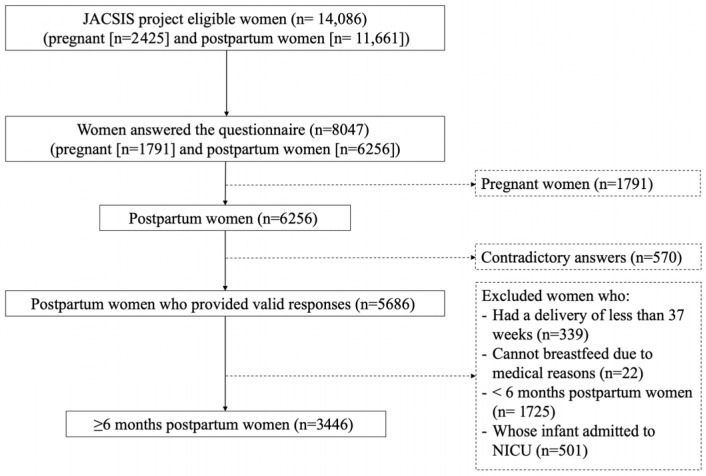
Enrollment of participants in this study.

The participant identification process commenced with an initial screening survey conducted in July 2021. This screening aimed to determine the eligibility of potential participants who had singleton births after July 2019, from which 11,661 postpartum mother-infant pairs were identified as potentially eligible. Subsequently, the internet research agency distributed an online questionnaire to all eligible postpartum mothers via an e-mail containing a link to a designated website. This effort yielded valid responses from 6256 postpartum women by 31 August 2021, corresponding to a response rate of 53.6%. Following data collection, we excluded 570 women who provided irrelevant or contradictory information on this survey [14]. In addition, we excluded postpartum women who had a delivery of less than 37 weeks (n = 139), who could not be followed due to medical reasons (n = 22), whose infants were younger than 6 months (n = 1725), or whose infants were admitted to a neonatal intensive care unit (NICU; n = 501). Finally, we obtained information from an anonymized dataset of 5686 postpartum mothers from all prefectures in Japan (Figure 1).

All individuals provided electronic informed consent before accessing the online questionnaire. The consent process fully informed participants about the nature of the study and their right to withdraw at any time. This research adhered strictly to the ethical principles outlined in the 1964 Declaration of Helsinki and its subsequent amendments. The research protocol was formally approved by the Institutional Review Board of the Osaka International Cancer Institute (Protocol Number 20084).

### 2.2. Measurements

#### 2.2.1. Exposure

The primary exposure was maternal ACEs, defined as distressing or traumatic events occurring before the age of 18. The assessment was based on a self-report questionnaire that inquired about participants’ retrospective experiences of childhood adversity. This study utilized a set of traditional ACE items [3] available in the 2021 survey, as the Japanese version of the Adverse Childhood Experiences Questionnaire (ACE-J) [15] was not administered in this analysis.

We evaluated nine specific types of adverse experiences. Each item was scored as a binary variable (1 = yes, 0 = no) to indicate the presence or absence of the experience. The nine items assessed were: (1) parental divorce or separation, (2) parental mental illness, (3) witnessing domestic violence, (4) physical abuse, (5) physical neglect, (6) psychological abuse, (7) sexual abuse, (8) experiences of poverty, and (9) bullying. We then created a cumulative ACE score by summing the affirmative responses for each participant, resulting in a score ranging from 0 to 9. This cumulative score quantifies the overall burden of childhood adversity. For the primary analysis, we categorized this score into a binary variable to compare mothers with a high burden of adversity (a score of 4 or more) against those with a lower burden (a score of 0 to 3). The original ACE study and its validation studies also suggested using a binary cutoff of ≥4 ACEs, as this number showed the strongest association with adverse health outcomes [3]. Although there is ongoing debate about using ACE scores as continuous vs. binary variables, the cutoff of 4 is commonly used because of its demonstrated association with higher health risks.

#### 2.2.2. Outcomes

The primary outcome was infant feeding behavior including breastfeeding, formula feeding, complementary foods. The study outcomes were derived from a primary questionnaire item that asked mothers about their infant feeding history: “Regarding your most recent child, from when and until when did you provide breast milk or infant formula?”. From the detailed responses to this question, we constructed several binary outcome variables for the analysis. For breastfeeding, the outcome ever breastfed was affirmative if the mother selected any response indicating she had provided breast milk, as opposed to selecting “have not given”. The variable late initiation of breastfeeding (≥7 days) was defined as affirmative if the mother reported starting to breastfeed one week or more after birth [2]. Similarly, continued breastfeeding (≥6 months) was considered affirmative if breastfeeding was maintained at six months postpartum or longer. Corresponding outcomes were created for formula feeding. The ever-used formula outcome was defined based on any reported use of infant formula [16]. We defined late initiation of formula feeding (≥7 days) as introducing formula one week or more after birth, and continued formula feeding (≥6 months) was affirmative if formula was still being used at six months of age or later [17]. The use of formula milk within the first week after birth often reflects early intervention due to insufficient breast milk production or medical indications (jaundice, hypoglycemia, etc.). On the other hand, delayed initiation after 7 days after birth may more strongly reflect maternal psychosocial factors and readiness for childcare, rather than acute problems [18]. Therefore, this study set this period as the cutoff value. Finally, based on a separate question about the timing of introducing complementary foods, we defined the outcome late initiation of complementary foods (≥7 months) as beginning to provide such foods at seven months of age or later. Our cutoff for late complementary feeding (≥7 months) aligns with WHO guidance recommending introduction at around 6 months [19]. These binary feeding outcomes served as the key dependent variables in the subsequent analyses.

#### 2.2.3. Other Variables

We collected data on several covariates. Basic and socioeconomic variables included maternal age (as a continuous variable), child’s sex (male or female), marital status (married or not), and living with a partner (yes/no). We also included maternal working status (working or not). Maternal education was classified into four groups: High school, Vocational school, Junior college, and University or graduate school. Household income was categorized into six groups: <2 million yen, ≥2 and <4 million yen, ≥4 and <6 million yen, ≥6 and <8 million yen, ≥8 and <10 million yen, and ≥10 million yen. We also gathered information on maternal health and perinatal and lifestyle factors. Maternal pre-pregnancy body mass index (BMI) (kg/m^2^) and gestational weeks at delivery were included as continuous variables. Parity was categorized as primipara or multipara. Low birth weight infants were defined as those with a birth weight less than 2500 g. Fertility treatments included timing, ovulation induction, artificial insemination of the husband, in vitro fertilization, and embryo transfer. For lifestyle factors, smoking and alcohol consumption were each categorized as “Current” or “Never/quit”. PPD was evaluated via the Edinburgh Postnatal Depression Scale (EPDS) and was divided into two groups: <9 and ≥9. Postpartum depression was measured using the EPDS, a widely used and validated screening instrument [20]. This questionnaire was answered by the women on the basis of information contained in the Maternal and Child Health Handbook provided by the local government to all pregnant women [21].

#### 2.2.4. Statistical Analyses

We performed descriptive analyses to summarize the characteristics of the mother–child pairs at first. We presented continuous variables as means with standard deviations (SD) and categorical variables as numbers with percentages (%). We describe the distribution of the cumulative ACE score (0–9) with mean, SD, median, IQR, and min–max and visualize it in a histogram (Figure S1). We also report floor/ceiling effects as the proportions scoring 0 and 9. We compared these baseline characteristics between mothers with low ACE scores (0–3) and those with high ACE scores (≥4) using Mann–Whitney U or *t*-tests for continuous variables; χ^2^ or Fisher’s exact test for categorical variables. To examine the association between maternal ACEs and infant feeding outcomes, we used logistic regression analysis. To control for confounding variables, we applied inverse probability of treatment weighting (IPTW), based on propensity scores. The propensity scores were calculated using seven maternal factors: age, pre-pregnancy body mass index (BMI), marital status, household income, parity, educational attainment, and living with a partner. For this analysis, the cumulative maternal ACE score was categorized as a binary variable (a score of ≥4 vs. 0–3). We calculated odds ratios (ORs) and their corresponding 95% confidence intervals (CIs) for each infant feeding outcome. To assess effect modification by PPD, we fitted IPTW-weighted logistic regression models including a product term for ACEs (≥4 vs. 0–3) × PPD (EPDS ≥ 9 vs. <9). We report Wald test *p*-values for interaction and provide stratum-specific adjusted odds ratios in Table S1. Reporting followed the STROBE guideline [22], and our analytic framework followed consensus references. Our use of propensity score–based inverse probability of treatment weighting (IPTW) to reduce confounding is consistent with methodological standards [23,24]. For effect-modification, we modeled a product term and reported Wald test *p*-values, following recommendations on presenting interaction analyses [25].

We also considered the potential role of intermediate factors, including maternal age (age ≥ 35 years or not) and household income (≥6 million yen or not). Stratified analyses were conducted to assess the effects of these factors separately. Furthermore, interaction terms were calculated for each stratified analysis to examine the influence of maternal ACEs on infant feeding outcomes in the presence of these intermediate factors. Because this was a secondary analysis of a fixed nationwide panel, it was not possible to perform an a priori sample-size calculation. To quantify the statistical potential of the available data, we approximated effective sample sizes (ESS) under IPTW within exposure strata (ACEs ≥ 4 vs. 0–3). The IPTW-weighted baseline risks in the reference group (ACEs 0–3) were 5.7% for late formula initiation (≥7 days) and 3.2% for late introduction of complementary foods (≥7 months). Using these risks and the adjusted odds ratios from the table in Section 3.2 (2.12 and 2.27, respectively), the achieved power at α = 0.05 (two-sided Pearson χ^2^ in PROC POWER, using group-specific ESS) was ≈77% for late formula initiation and ≈67% for late complementary foods. A simple grid check further indicated that, under the observed ESS and baseline risks, smaller effects would be markedly underpowered, and that an effect size larger than the observed estimates would be required to ensure ≥80% power. All the statistical analyses were performed via SAS software (version 9.4; SAS Institute Inc., Cary, NC, USA).

## 3. Results

### 3.1. Participant Characteristics

This study included 3446 postpartum mothers. Of these, 3225 (93.6%) mothers reported 0–3 ACEs, while 221 (6.4%) reported 4 or more ACEs. Mothers with 4 or more ACEs were more likely to be older, have lower BMI, marriage, and education level compared to those with 0–3 ACEs. The high-ACE group also reported lower household incomes, with a higher percentage earning less than 4 million yen annually and less living with a partner. Furthermore, mothers with 4 or more ACEs had a higher prevalence of current smoking and were significantly more likely to experience PPD (Table 1).

### 3.2. Association Between Maternal ACEs and Infant Feeding Outcomes

As shown in Table 2, high maternal ACEs (≥4) were significantly associated with delayed infant feeding initiation. Mothers with 4 or more ACEs significantly delayed the initiation of formula feeding (≥7 days) (Adjusted OR: 2.12, 95% CI: 1.12–4.01, *p* = 0.02) and complementary foods (≥7 months) (Adjusted OR: 2.27, 95% CI: 1.38–5.01, *p* = 0.03) compared to mothers with 0–3 ACEs. There were no statistically significant associations observed for other outcomes, including ever breastfeeding, late initiation of breastfeeding, continued breastfeeding (≥6 months), ever using formula, or continued formula feeding (≥6 months) (Table 2).

### 3.3. Mediating Role of Postpartum Depression, Smoking, and Alcohol

The association between high maternal ACEs and late initiation of formula feeding was no longer statistically significant after adjusting for PPD (Adjusted OR: 1.77, 95% CI: 0.92–3.37, *p* = 0.09). Similarly, the association with late introduction of complementary foods was attenuated and lost statistical significance after adjusting for PPD (Adjusted OR: 2.15, 95% CI: 0.97–4.76, *p* = 0.06).

In contrast, adjusting for maternal smoking or alcohol consumption individually did not meaningfully alter the associations with delayed feeding initiation. Furthermore, when all three potential mediators were included in the model, the associations with both delayed feeding outcomes became non-significant (Table 3).

## 4. Discussion

This study, utilizing a large nationwide Japanese dataset, provides crucial insights into the relationship between maternal ACEs, PPD, and the timing of infant feeding initiation. Our principal finding is that a high maternal ACE score (four or more) is significantly associated with a delayed start to both formula feeding (≥7 days) and the introduction of complementary foods (≥7 months). Notably, this association was not observed for breastfeeding-related outcomes. The most significant finding of this study is that these associations with delayed feeding were attenuated to non-statistical significance after adjusting for maternal PPD, suggesting that PPD is a powerful mediator in the pathway from maternal ACEs to delays in infant feeding behaviors.

Several mechanisms could explain how maternal ACEs lead to these delays. First, chronic stress from childhood adversity is known to impair executive functions, such as planning, problem-solving, and emotional regulation [26]. The tasks of preparing formula or introducing complementary foods require significant planning and effort, which may be challenging for mothers whose cognitive resources are compromised by the long-term effects of trauma. Second, a history of ACEs can decrease a mother’s mental and physical energy, leading to reduced motivation and engagement in parenting tasks that are not perceived as immediately essential for survival [27,28]. Operating in a constant state of high alert or “survival mode” consumes significant cognitive and emotional bandwidth, leaving little reserve for other tasks. For a mother who is already overwhelmed, the complex and novel process of introducing solids may feel like an insurmountable demand, prompting her to stick with the simpler, more predictable routine of bottle-feeding. Third, mothers with a history of ACEs may face barriers to accessing healthcare and social resources, potentially missing timely guidance on infant nutrition [29]. These barriers are not simply practical; they are often psychological, rooted in a distrust of institutions or authority figures stemming from past trauma. A fear of being judged as a “bad mother,” combined with potential social isolation, can create a profound reluctance to seek help. Therefore, these mechanisms suggest it is crucial to intervene not just by providing nutritional information, but by focusing on alleviating the mother’s psychological burden and building trusting, relationship-based support systems that offer practical and continuous parenting guidance.

This study’s results strongly indicate that PPD is a primary pathway through which maternal ACEs affect infant feeding. The link between ACEs and an elevated risk for PPD is well-established in the literature, a finding our data corroborates, showing that mothers in the high-ACE group had a significantly higher prevalence of PPD [7,30,31]. The core symptoms of PPD—including anhedonia, profound fatigue, and difficulty concentrating—can directly decrease a mother’s capacity to engage in the demanding tasks of parenting. Thus, our findings suggest that the impact of maternal ACEs on feeding initiation often operates by first increasing a mother’s vulnerability. This suggests that the effect is indirect; maternal ACEs do not directly cause delays, but rather operate through the pathway of PPD, where symptoms like anhedonia, profound fatigue, and difficulty concentrating directly impair a mother’s capacity for complex parenting tasks. This reframes the primary intervention target from addressing past trauma to alleviating current depressive symptoms. Effectively treating PPD is therefore a tangible strategy to facilitate timely infant feeding. Nevertheless, it would be an oversimplification to view PPD as the sole mediator. The pathway is likely more complex, with other factors such as anxiety disorders (which are highly comorbid with PPD), low social support, or the mother’s own attachment style also contributing to this relationship [32]. PPD may be a critical and measurable component, but it is part of a larger constellation of psychosocial challenges that follow from a history of ACEs.

The outcome definition used in this study, particularly defining the late introduction of complementary foods as initiation at or after seven months of age, is clinically relevant and robust. World Health Organization (WHO) consistently recommends the introduction of complementary foods at approximately six months of age to meet the infant’s evolving nutritional and developmental needs [19]. A large-scale Korean study of 206,248 children found that introducing complementary foods at or after 7 months of age was associated with more frequent hospitalizations and an increased risk of low height-for-age [12]. Therefore, defining “delay” as initiation at seven months or later captures a group of infants who have clearly missed this recommended window. This affirms that the association found in our study represents a clinically meaningful delay that could have tangible consequences for infant nutrition and development, elevating it from a mere statistical observation to a public health concern.

Previous studies have reported the impact of ACEs on infant feeding behavior. A retrospective study of 926 women in Northern California revealed that those with 2 more ACEs had increased odds of any breastfeeding and continued exclusive breastfeeding at 2 months postpartum [10]. Another Canadian population-based study found that while ACE exposure did not affect breastfeeding initiation, it was associated with a lower likelihood of exclusive breastfeeding for up to six months [11]. However, other studies have not found a direct relationship between high maternal ACE scores and breastfeeding behaviors or infant growth, highlighting the role of resilience and broader contextual factors [33]. Some studies theorize that mothers with ACEs may initially overcompensate in their parenting—making greater efforts to breastfeed as a way to forge strong attachment—though sustaining breastfeeding can be more challenging, possibly due to stress or lack of ongoing support. Most literature focuses on breastfeeding, with less direct evidence regarding how maternal ACEs influence the timing or pattern of introducing complementary foods or formula specifically, and the direct impact of maternal ACEs on these choices remains less studied.

This study has several limitations that must be acknowledged. First, its cross-sectional design prevents the establishment of definitive causal relationships. Second, our data collection method has inherent limitations. The reliance on self-report questionnaires may introduce recall bias, and the online survey format, with a response rate of 53.6%, may introduce selection bias. Third, the ACEs assessment used a cumulative score from available items rather than a standardized scale like the ACE-J, which may limit comparability. Crucially, this cumulative approach treats all ACEs as equal in weight, masking the potential for different types of adversity to have different effects. Other research, for instance, has found that specific experiences like neglect versus abuse may have unique impacts on later outcomes, a nuance our study could not explore. Fourth, despite using IPTW, residual confounding from unmeasured variables—such as partner support or infant temperament—may remain. Despite using IPTW, residual confounding from unmeasured variables—such as partner support or infant temperament—may remain. In addition, although the main IPTW models were adequately powered for moderate-to-large effects (≈77% for late formula initiation and ≈67% for late complementary feeding), the effective sample sizes under weighting limited the ability to detect smaller effects, and exploratory stratified or interaction analyses should be interpreted with caution. Finally, data were collected during the COVID-19 pandemic, a unique stressor that may affect the generalizability of our findings.

Our findings support routine PPD screening (EPDS) and targeted counseling among mothers with high ACE exposure, integrated into well-child and postpartum visits. Beyond nutrition education, trauma-informed, relationship-based support (e.g., antenatal/postnatal counseling embedded in perinatal care) may address psychosocial barriers that delay feeding transitions. Aligning with the WHO guidance on complementary feeding around 6 months, clinicians should prioritize timely, staged guidance for at-risk mothers.

## 5. Conclusions

In conclusion, this study demonstrates that maternal ACEs are a significant risk factor for delays in the initiation of formula and complementary feeding in infants. Crucially, this relationship appears to be largely mediated by PPD. These findings have important clinical and public health implications. Clinically, they underscore the need to screen for a history of ACEs during prenatal care. Mothers with high ACE scores should be considered a high-risk group for PPD, warranting enhanced screening and proactive mental health support throughout the perinatal period. From a public health perspective, our results suggest that interventions aimed at promoting timely infant feeding should not focus solely on nutritional education but must also integrate maternal mental health support. Adopting trauma-informed care approaches within maternal and child health programs is essential to create a safe and supportive environment for parents with a history of adversity. Future research should employ longitudinal designs to further clarify these causal pathways and test interventions—particularly antenatal and postnatal counseling embedded within trauma-informed perinatal care—aimed at mitigating PPD to see if they can, in turn, prevent delays in infant feeding initiation and improve child health outcomes.

## Figures and Tables

**Table 1:** Characteristics of mother–child pairs according to maternal adverse childhood experiences.

		ACE 0–3	ACE 4 Over	*p*-Value
N (%)		3225 (93.6)	221 (6.4)	―
Maternal age (years ± SD)		32.4 ± 4.3	32.5 ± 4.9	0.007
Pre-pregnancy body mass index (kg/m^2^ ± SD)		23.3 ± 17.8	22.9 ± 13.8	<0.001
Child’s sex (boy, %)		1578 (48.9)	117 (52.9)	0.25
Marital status (marriage, %)		3183 (98.7)	210 (95.0)	<0.001
Gestational weeks (weeks ± SD)		39.1 ± 1.2	39.1 ± 1.8	0.10
Maternal education, n (%)				<0.001
	High school	449 (14.0)	81 (36.8)	
	Vocational school	612 (19.0)	53 (24.1)	
	Junior college	405 (12.6)	23 (10.5)	
	University or graduate school	1750 (54.4)	63 (28.6)	
Household income, n (%)				0.005
	<2 million yen	51 (1.6)	10 (4.5)	
	≥2 and <4 million yen	255 (7.9)	26 (11.8)	
	≥4 and <6 million yen	759 (22.5)	56 (25.3)	
	≥6 and <8 million yen	779 (24.2)	49 (22.2)	
	≥8 and <10 million yen	503 (15.6)	27 (12.2)	
	≥10 million yen	1023 (27.2)	53 (24.0)	
Living with a partner, n (%)		3149 (97.6)	207 (93.7)	0.002
Working, n (%)		2111 (82.4)	134 (80.7)	0.59
Fertility treatments, n (%)		372 (11.5)	20 (9.1)	0.26
LBWI, n (%)		186 (5.8)	6 (2.7)	0.06
Mode of delivery, n (%)				0.43
Spontaneous		2668 (82.7)	183 (82.8)	
Cesarean delivery		557 (17.3)	38 (17.2)	
Parity				0.23
Primipara		1753 (54.4)	111 (50.2)	
Multipara		1472 (45.6)	110 (49.8)	
Smoking, n (%)				<0.001
Never/quit		3077 (95.4)	195 (88.2)	
Current smoking		148 (4.6)	26 (11.8)	
Alcohol consumption, n (%)				0.09
Never/quit		2372 (73.5)	151 (68.3)	
Current smoking		853 (26.5)	70 (31.7)	
Postpartum depression				<0.001
EPDS score < 9		2435 (75.5)	120 (54.3)	
EPDS score ≥ 9		790 (24.5)	101 (45.7)	

ACEs, adverse childhood experiences; LBWI, low birth weight infant; EPDS, Edinburgh Postnatal Depression Scale.

**Table 2:** Association between maternal adverse childhood experiences (ACEs) and infant feeding outcomes.

		Crude OR (95% CI)	Adjusted OR (95% CI)	*p*-Value	Adjusted *p*-Value
*Breastfeeding*					
	Ever breastfed				
	ACE 0–3 (reference)	1.00	1.00		
	ACE 4 over	0.18 (0.05–0.69)	0.31 (0.07–1.47)	0.01	0.14
	Late initiation of breastfeeding (≥7 days)				
	ACE 0–3 (reference)	1.00	1.00		
	ACE 4 over	2.62 (1.08–6.35)	2.27 (0.92–5.56)	0.03	0.08
	Continued breastfeeding (≥6 months)				
	ACE 0–3 (reference)	1.00	1.00		
	ACE 4 over	1.14 (0.71–1.84)	1.07 (0.67–1.72)	0.58	0.77
*Formula feeding*					
	Ever used formula				
	ACE 0–3 (reference)	1.00	1.00		
	ACE 4 over	1.79 (0.56–5.75)	0.94 (0.40–2.23)	0.33	0.90
	Late initiation of formula feeding (≥7 days)				
	ACE 0–3 (reference)	1.00	1.00		
	ACE 4 over	1.82 (0.92–3.63)	2.12 (1.12–4.01)	0.09	0.02
	Continued formula feeding (≥6 months)				
	ACE 0–3 (reference)	1.00	1.00		
	ACE 4 over	0.96 (0.64–1.44)	0.99 (0.68–1.49)	0.85	0.97
*Complementary feeding*					
	Late initiation of complementary foods (≥7 months)				
	ACE 0–3 (reference)	1.00	1.00		
	ACE 4 over	2.20 (0.97–4.96)	2.27 (1.38–5.01)	0.16	0.03

OR, odds ratio; CI, confidence interval; ACE, adverse childhood experiences. Adjusted odds ratios were estimated by logistic regression with inverse probability of treatment weighting (IPTW), based on a propensity score adjusted for maternal age, pre-pregnancy BMI, marital status, household income, parity, maternal education, and living with a partner.

**Table 3:** Association between ACEs and infant feeding outcomes stratified by smoking, alcohol, and PPD.

		Adjusted OR (95% CI)	*p*-Value
Late initiation of formula feeding (≥7 days)			
	Model 1: Main Model (from Table 2)	2.12 (1.12–4.01)	0.02
	Model 2: Main Model + PPD	1.77 (0.92–3.37)	0.09
	Model 3: Main Model + Smoking	2.13 (1.12–4.04)	0.02
	Model 4: Main Model + Alcohol consumption	2.11 (1.12–4.00)	0.02
	Model 5: Main Model + All mediators (PPD, Smoking, Alcohol)	1.79 (0.93–3.43)	0.08
Late introduction of complementary foods (≥7 months)			
	Model 1: Main Model (from Table 2)	2.27 (1.38–5.01)	0.03
	Model 2: Main Model + PPD	2.15 (0.97–4.76)	0.06
	Model 3: Main Model + Smoking	2.28 (1.04–4.98)	0.04
	Model 4: Main Model + Alcohol consumption	2.34 (1.07–5.13)	0.03
	Model 5: Main Model + All mediators (PPD, Smoking, Alcohol)	2.21 (0.99–4.89)	0.05

Abbreviations: OR, odds ratio; CI, confidence interval; ACE, adverse childhood experiences; PPD, postpartum depression. All models are adjusted using IPTW based on the propensity score from Table 2. Model 1 represents the main adjusted model. Models 2 through 5 show the results after additionally adjusting for postpartum depression (PPD), smoking, alcohol consumption, and all mediators combined to assess their impact on the odds ratios.

## Data Availability

Owing to the presence of personally identifiable or potentially sensitive information, the data used in this study were not deposited in a public repository. The Research Ethics Committee of the Osaka International Cancer Institute restricted data sharing in compliance with Japanese ethical guidelines. All questions regarding data use can be directed to Takahiro Tabuchi at tabuchitak@gmail.com. Additional information on data availability can be obtained from the JACSIS website (https://jacsis-study.jp/dug/index.html).

## Supplementary Materials

The following supporting information can be downloaded at: https://www.mdpi.com/article/10.3390/nu17172879/s1, Figure S1. Histogram of the cumulative ACE score (0–9); Table S1: Effect modification analysis of maternal ACEs and delayed infant feeding outcomes by postpartum depression.

## Abbreviations

NICU	Neonatal intensive care unit
ACEs	Adverse childhood experiences
JACSIS	Japan COVID-19 Social Internet Survey
OR	Odds ratio
CI	Confidence interval
ACE-J	Japanese-adapted version of the Adverse Childhood Experiences Questionnaire
IPTW	inverse probability of treatment weighting
BMI	Body mass index
SD	Standard deviations
EPDS	Edinburgh Postnatal Depression Scale
