# Supplementary material for: Maternal Adverse Childhood Experiences and Delayed Initiation of Complementary Foods: A Nationwide Online Cohort Study
Source: Nutrients. 2025 Sep 5;17(17):2879. doi: 10.3390/nu17172879 (PMC12430508; doi:10.3390/nu17172879)
Supplement: Supplementary file 1 [file nutrients-17-02879-s001.zip › nutrients-3838020-supplementary.pdf]

Supplementary Materials

Figure S1. Histogram of the cumulative ACE score (0–9)

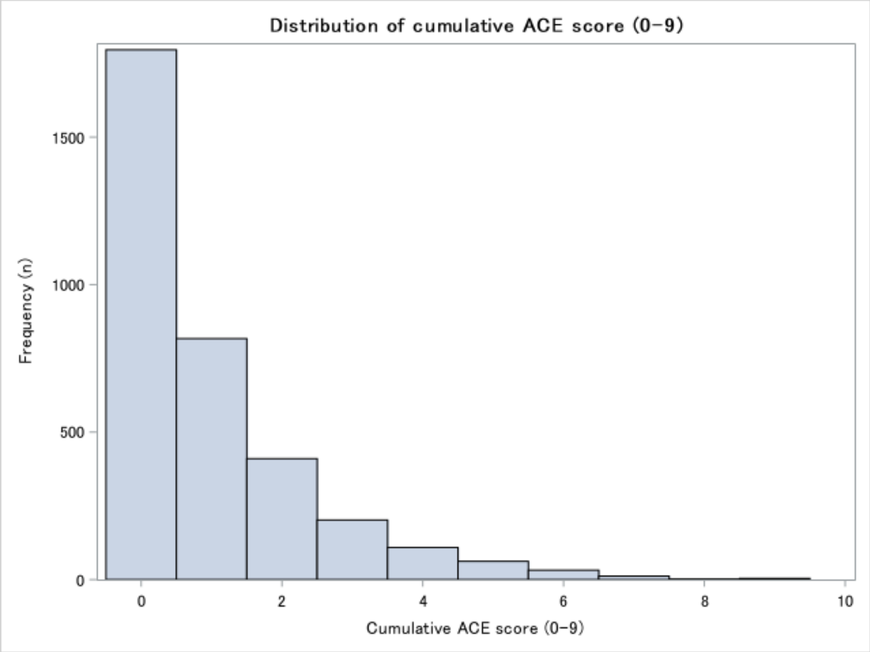

Note: The distribution of cumulative ACE scores was skewed toward the lower end, with a mean (SD) of 0.96 (1.38), median of 0 (IQR 0–1), and a range of 0–9. Nearly all respondents scored 0 (99.8%), while only 0.2% scored 9.

Table S1. Effect modification analysis of maternal ACEs and delayed infant feeding outcomes by postpartum depression.

	Interaction p (Wald)	PPD stratum	Adjusted OR (95%CI)
Late initiation of formula feeding (≥ 7days)	0.21	PPD=0	1.68 (0.71-3.95)
		PPD=1	1.88 (0.70-5.07)
Late introduction of complementary foods (≥ 7months)	0.08	PPD=0	1.80 (0.54-5.99)
		PPD=1	2.49 (0.88-7.10)

Notes: Interaction p-values are derived from IPTW-weighted logistic regression models including maternal ACEs (≥4 vs. 0–3), postpartum depression (PPD, EPDS ≥9 vs. <9), and their product term (ACEs × PPD). Stratum-specific adjusted odds ratios (aORs) were estimated from IPTW-weighted logistic models fitted separately within PPD strata, with ACE (high vs. low ACEs) as the exposure. Although point estimates were consistently above 1, 95% confidence intervals crossed 1, reflecting reduced precision after stratification and weighting. These results should therefore be interpreted as exploratory.
